# Balancing in a black box: Potential immunomodulatory roles for TGF-β signaling during blood-stage malaria

**DOI:** 10.1080/21505594.2020.1726569

**Published:** 2020-02-11

**Authors:** Lisa L. Drewry, John T. Harty

**Affiliations:** a Department of Microbiology and Immunology, University of Iowa, Iowa City, IA, USA; b Department of Pathology, University of Iowa, Iowa City, IA, USA; c Interdisciplinary Graduate Program in Immunology, University of Iowa, Iowa City, IA, USA

**Keywords:** Malaria, TGF-β, inflammation

## Abstract

Malarial disease caused by *Plasmodium* parasites challenges the mammalian immune system with a delicate balancing act. Robust inflammatory responses are required to control parasite replication within red blood cells, which if unchecked, can lead to severe anemia and fatality. However, the same inflammatory response that controls parasite replication is also associated with immunopathology and severe disease, as is exemplified by cerebral malaria. A robust literature has identified critical roles for innate, cellular, and humoral immune responses orchestrated by IFN-γ and T_H_1 type responses in controlling blood stage malarial disease. In contrast, TGF-β and IL-10 have been identified as important anti–inflammatory immunomodulators that help to limit inflammation and pathology during malaria. TGF-β is a pleiotropic cytokine, with the ability to exert a wide variety of context-dependent immunomodulatory roles.

The specific mechanisms that allow TGF-β to protect against malarial pathology remain essentially unexplored and offer a promising avenue to dissect the most critical elements of immunomodulation in avoiding severe malaria. Here we discuss potential immunomodulatory roles for TGF-β during malaria in light of recent advances in our understanding of the role of Tregs during blood-stage malaria.

## Malarial disease

Malarial disease imposes a severe burden on global health, causing over 200 million annual cases [1]. Anti-malarial therapeutics are available. However, disease mortality is still high, with over 400,000 malaria deaths estimated in 2018 [1]. The RTS,S/AS01 Mosquirix^TM^ vaccine was recently approved for use in pilot childhood immunization programs in selected regions of three endemic countries, but shows only limited and short-term efficacy [2–5]. Moreover, parasite resistance to all available anti-malarial therapeutics further threatens malaria control efforts. Improved therapeutics and vaccines for malaria are thus direly needed.

Malaria is caused by infection with *Plasmodium* parasites. Parasites cycle between *Anopheline* mosquito vectors and mammalian hosts. Infection in mammals is initiated upon the bite of gravid mosquitoes, which transfers the sporozoite form of *Plasmodium* parasites into the host dermis. *Plasmodium* sporozoites migrate through the skin and eventually enter blood vessels [6]. By traveling through the blood circulation, sporozoites reach the liver, where they traverse and then eventually invade into hepatocytes. Within hepatocytes, parasites massively replicate into large schizont stages. Upon completion of hepatic replication, schizonts rupture and release merosomes that contain merozoites, thousands of which can be produced by a single infected hepatocyte [7–9]. Upon release from merosomes, merozoites go on to invade red blood cells (RBCs). Merozoite invasion of and amplification within RBCs is responsible for the clinical symptoms of malaria [10]. In contrast, the liver stage of infection is clinically silent. However, parasite burden in the liver is typically low, making it an appealing target for prophalytic interventions such as vaccines [11,12].

Most severe malaria in humans is caused by *Plasmodium falciparum*. However, *Plasmodium vivax* also causes a substantial number of typically uncomplicated but persistent cases, and limited numbers of cases are also caused by *Plasmodium ovale, Plasmodium malariea* and *Plasmodium knowlesi* [10]. The hallmark presentation of malaria is cyclical fevers. Further nonspecific symptoms include headache, stiffness, nausea, and muscle pain [10]. In uncomplicated malaria, symptoms subside as blood parasitemia is controlled, but both illness and parasitemia will periodically rebound in cycles of typically decreasing severity [10].

Individuals residing in endemic regions experience repeated exposures and develop a clinical immunity that suppresses parasite replication, but often allows for low-level asymptomatic blood-stage infections to persist [13]. Infections with *P. vivax* can also relapse upon activation of hypnozoites, a dormant liver stage of this *Plasmodium* species [14]. Individuals that fail to efficiently control the parasite burden and immunopathology of malaria experience severe disease. Malaria fatalities generally stem from severe anemia, cerebral malaria, or respiratory distress. The pathogenesis underlying severe disease is still incompletely understood. However, rapid parasite proliferation, unconstrained inflammatory responses, and microvascular obstruction are generally agreed to be the major causes of severe disease [10].

*Plasmodium* parasites encounter robust innate and adaptive immune responses at all stages of infection in humans. However, in humans the immune response struggles to efficiently clear primary infections, instead allowing infections to persist as long-term low-level asymptomatic blood-stage infections, which can recrudesce repeatedly before robust control is achieved. The resulting immunity also fails to provide sterilizing protection against subsequent re-infection. Instead, with repeated exposures, a clinical immunity is generated in which symptoms of anemia and fever are mild or absent, but blood-stage infection and transmission continue [10].

Controlling malaria challenges the host immune system with a high-stakes balancing act. Robust inflammatory responses are required to control parasite replication. In particular, humoral immunity orchestrated by CD4 T cell-dependent antibody responses controls parasitemia and thus allows for the resolution of blood-stage disease. The pro-inflammatory cytokines interferon gamma (IFN-γ), tumor necrosis factor alpha (TNF-α), and interleukin 12 (IL-12) are crucial mediators of the anti-parasitic response. However, high levels of inflammation are also linked to the pathology of severe malaria [15,16]. Accordingly, immunomodulatory cytokines including transforming growth factor beta (TGF-β) and interleukin 10 (IL-10) are theorized to fine tune the extent of inflammation so that parasite burden is controlled while inflammatory pathology is minimized [15]. Although several lines of evidence support the ability of TGF-β to modulate excessive inflammation during malaria, the mechanistic basis by which TGF-β protects against immunopathology remains largely unexplored [17].

## The pleiotropic immunomodulatory cytokine, TGF-β

TGF-β is a pleiotropic cytokine that plays pivotal roles in immunomodulation. Three TGF-β isoforms are expressed by mammals, but the primary isoform expressed in immune cells is TGF-β1 [18]. TGF-β is produced as a latent pro-protein harboring an N-terminal peptide that is removed by a furin-like peptidase in the Golgi. This cleavage event produces the latency-associated protein (LAP), which non-covalently associates with mature TGF-β and prevents receptor binding. The mature TGF-β LAP complex is termed the small latent complex (SLC) and forms a further complex, the large latent complex (LLC), through covalent binding of the LAP with the large latent TGF-β -binding protein (LTBP). Efficient incorporation of the LTBP into extracellular matrix (ECM) allows stable storage of LLCs harboring latent TGF-β within ECM for subsequent activation [19].

Engagement of LTBP with RGD integrin motifs releases LAP and allows TGF-β to bind a tetrameric receptor complex composed of two molecules each of the receptors TGF-βRI and TGF-βRII. Both receptors are serine/threonine kinases. TGF-β engagement of the TGF-βRI–II complex initiates a signaling cascade characterized by activation of Smad transcription factors [19]. Smads directly bind DNA, but work in cooperation with further transcription factors and co-regulators. The cooperative nature of Smad activity allows Smads to exert a variety of activating and repressive effects on gene expression, depending on the cellular and molecular environment provided [19]. Receptor engagement by TGF-β also induces several Smad-independent signaling pathways, such as PI3K-Akt and mitogen-associated protein (MAP) kinase [19,20]. Accordingly, TGF-β signaling can exert pleiotropic effects that are highly context-dependent.

TGF-β regulates the development, maintenance, and activity of many immune cells. Deletion of *Tgfb1* in mice leads to a lethal inflammatory response [21,22], underscoring the importance of TGF-β for successful modulation of immunity. T cells are well established as critical negatively regulated targets of TGF-β signaling, as T-cell specific deletion of TGF-βRII phenocopies the lethal inflammatory defect of *Tgfb1* knockout mice [18]. TGF-β signaling inhibits differentiation of T helper 1 (T_H_1), T helper 2 (T_H_2), and cytotoxic T-lymphocytes (CTLs), and promotes differentiation of T helper 17 (T_H_17), T helper 9 (T_H_9), T follicular helper cells (TFH), and Tregs [18,23]. In B cells, TGF-β inhibits proliferation, survival, and IgG class switching, but promotes IgA production. TGF-β also inhibits natural killer cell (NK) development and function [23].

Unsurprisingly given its pleiotropic nature, TGF-β influences parasitic infections in a variety of manners. TGF-β activity enhances the virulence of the protozoan parasites *Trypanosoma cruzi* and *Leishmania braziliensis* by enabling more robust growth within macrophages [24,25]. The helminth worm *Heligmosomoides polygyrus* also reaches higher burdens with intact TGF-β signaling [26]. Production of TGF-β by resident intestinal intraepithelial CD8 T cells was critical for controlling immunopathology during intestinal infection with the protozoan parasite *Toxoplasma gondii* [27].

## TGF-β and human malaria

The involvement of TGF-β in human malaria has been investigated with epidemiological approaches and controlled human malaria infection (CHMI) studies. CHMI studies where malaria-naïve volunteers were challenged with *P. falciparum* sporozoites noted that subjects segregated into distinct patterns of cytokine production during the innate response to acute infection [28,29]. In groups where TGF-β production was more robust, subjects experienced less disease pathology, which was accompanied by higher parasitemia [28,29]. This would suggest that a response where TGF-β is abundant is associated with less severe malaria pathology. However, longitudinal analysis of children in endemic regions has shown that recent *Plasmodium* infection clearly alters the cytokines produced during innate response to acute infections [13], an issue that needs to be considered when comparing results from CHMI studies conducted with malaria-naïve subjects to infections as they naturally occur in endemic regions.

Multiple epidemiological case-control studies have also identified an inverse correlation between TGF-β abundance and occurrence of severe or complicated disease during *P. falciparum* infections in malaria-endemic regions [30–35]. Adult patients treated for symptomatic *P. falciparum* malaria in Bangkok, Thailand were found to exhibit diminished levels of serum TGF-β relative to healthy controls [31]. In this study, serum TGF-β levels negatively correlated with the abundance of TNF-α, a pro-inflammatory cytokine elevated in human cerebral malaria patients and during experimental cerebral malaria in mice [16,31,36]. To further probe a potential connection between TGF-β and the development of severe or complicated disease, several studies stratified subjects into mild, severe, and complicated malaria presentations. In Ugandan children, serum TGF-β content was significantly diminished in uncomplicated malaria cases relative to healthy controls, and even further decreased in cerebral malaria cases [30]. Among the severe malaria cases, TGF-β content was inversely correlated with the pro-inflammatory cytokines IFN-γ and IL-6, but perplexingly also the anti–inflammatory cytokine IL-10 [30]. Separate studies in Tanzania and Thailand found that TGF-β abundance was more strongly decreased in cases of severe and cerebral malaria than in uncomplicated malaria [32]. Mozambican children were also reported to exhibit subtly less elevated TGF-β in cases of severe malaria, compared to cases of uncomplicated malaria [35]. These findings are generally consistent with a model where TGF-β limits the extent of pathology associated with malaria disease in endemic regions.

Unsurprisingly given the diverse presentation of human malaria, not all studies have suggested robust pathology control by TGF-β. In one study performed in Ghana, a high ratio of TGF-β to the pro-inflammatory cytokines IFN-γ and TNF-α correlated with increased risk of fever, but not clinical malaria presentation [34]. Another study assessing Gabonese children reported that TGF-β levels were significantly diminished in children experiencing both mild and severe malaria relative to healthy controls, but TGF-β abundance was not significantly more diminished in severe malaria cases relative to mild malaria [33]. Most strikingly, a study in Assam, India, reported that TGF-β expression was enhanced in both *P. falciparum*-infected humans relative to healthy controls, and complicated malaria cases relative to uncomplicated malaria [37].

In aggregate, epidemiological studies of human malaria suggest that TGF-β immunomodulation during malaria may be protective against severe disease. Discrepancies among some of the studies are likely due to the influence of extensive variability in genetics, nutrition, infection history, and co-infection status among infected humans, as well as genetic differences among circulating *Plasmodium* parasites in various endemic regions. However, it still remains uncertain whether TGF-β production causally contributes to moderating disease severity, or is simply a biomarker of a less intense infection that does not produce pathology because an extreme inflammatory response is not required to control parasite replication. Murine malaria models have been used to investigate these questions with a less variable system that is amenable to experimental manipulation, and support a protective and time-sensitive role for TGF-β during blood-stage infection.

## Investigating roles for TGF-β during malaria with murine models

A collection of *Plasmodium* parasite species that naturally infect African thicket rats are amenable to experimental infection of laboratory mice, and cause a spectrum of malaria disease that approximates many features of human malaria [38].

*P. yoelli, P. chabaudi,* and *P. berghei* are the most commonly used parasites in murine malaria studies. Some strains of *P. yoelli* (for example, the widely used strain 17XNL) and *P. chabaudi* (for example, strain AS) cause resolving, typically non-lethal malaria disease in mice that is characterized by anemia and hypoglycemia reminiscent of human malaria [38,39]. During *P. chabaudi* malaria, antibody-mediated defenses suppress an initial peak of parasitemia to low levels, but parasites recrudesce several times before final clearance. *P. chabaudi* malaria also features endothelial sequestration of infected RBCs, although this is concentrated in the liver and lung rather than the brain as in human cerebral malaria. In contrast to the fever characteristic of human malaria, *P. chabaudi* malaria causes hypothermia in mice [38]. During *P. yoelli* malaria, antibody-mediated defenses also suppress an initial parasitemia peak, typically clearing the infection. Recrudescence after primary clearance and RBC sequestration are not typically observed in *P. yoelli* malaria [38,39]. More severe malaria that includes higher parasitemia and fatality can be studied in a lethal strain of *P. yoelli*, 17XL, which efficiently invades non-reticulocyte RBCs due to a mutated parasite erythrocyte binding ligand (EBL) [40]. The ANKA strain of *P. berghei* has been extensively used in the experimental cerebral malaria (ECM) model of human cerebral malaria. In ECM, *P. berghei* ANKA infection of mice of varying susceptibility (C57BL/6, CBA/J, and Swiss Webster – highly susceptible; BALB/c and A/J- relatively resistant) causes neurological symptoms similar to cerebral malaria in humans [41]. How effectively ECM models the etiology of human cerebral malaria has been robustly debated [36,42,43], as sequestration of infected RBCs in the brain is characteristic of human cerebral malaria but less pronounced during ECM. In all murine models, infection resolution generally results in protection against re-challenge by the same *Plasmodium* species [39]. This contrasts the susceptibility of malaria-experienced humans to re-infection, which can instead be modeled by challenge of malaria-experienced mice with heterologous *Plasmodium* species, to which they are typically susceptible [39].

Early work used comparative analyses of these *Plasmodium*-species capable of murine infection to theorize roles for TGF-β during malaria. In a *P. berghei* ANKA infection model of ECM, susceptible CBA/J mice expressed less TGF-β and more IFN-γ than resistant BALB/c mice [44]. Further work compared mice infected with *Plasmodium* parasites of varying virulence. TGF-β levels were low in lethal infections with *P. berghei* NK65 and higher in resolving infections with *P. chabaudi* or *P. yoelli (*strain 17XNL) [45]. These results pointed to a model where TGF-β production helps to limit disease severity in resolving infections. Conversely, insufficient TGF-β production would allow an unchecked anti-parasitic inflammatory response to cause pathology and lethal outcomes. Consistent with this model, administering recombinant TGF-β to mice infected with typically lethal *P. berghei* NK65 prolonged mouse survival [45]. Neutralizing TGF-β also hastened mortality in *P. berghei* NK65 infections and converted typically resolving *P. chabaudi* infections into lethal infections [45]. However, neutralization of TGF-β during *P. yoelli* 17XNL infection did not similarly aggravate mortality [45]. This observation could be explained by compensatory increases in other anti–inflammatory cytokines, such as was later reported to occur upon TGF-β neutralization during infection with the closely related strain *P. yoelli* 17XL [46], or could suggest that TGF-β activity is higher than optimal during *P. yoelli* 17XNL infection.

Subsequent investigations showed the importance of the timing and magnitude of TGF-β activity during murine malaria. Comparison of the lethal (17XL) and non-lethal (17XNL) variants of *P. yoelli* demonstrated that *P. yoelli* 17XL infection was characterized by a very early burst of TGF-β, detectable within 24 hours of infection [46]. The early TGF-β burst in 17XL infected mice correlated with diminished early production of the pro-inflammatory cytokines IFN-γ and TNF-α, relative to 17XNL infections [46]. Neutralizing TGF-β did not alter the course of *P. yoelli* 17XL infection, likely because of compensatory increases in the anti–inflammatory cytokine IL-10. However, dual neutralization of TGF-β and IL-10 increased early IFN-γ and TNF-α production, diminished peak parasitemia, and prolonged mouse survival [46]. Unlike the protective effects of TGF-β during *P. chabaudi* infection, the rapid and early TGF-β burst during *P. yoelli* 17XL malaria thus appears to favor the parasite and harm the host. Rather than dampen an over-exuberant inflammatory response, early TGF-β production during 17XL infection limits an early inflammatory response critical for controlling parasite replication. Not surprisingly, extreme blunting of the anti-malarial inflammatory response by TGF-β can also lead to poor outcomes in resolving infections. Administration of high levels of recombinant TGF-β suppressed IFN-γ production in C57BL/10 mice infected with *P. chabaudi*, leading to a lethal outcome in this typically resolving infection [47].

**Figure 1.**
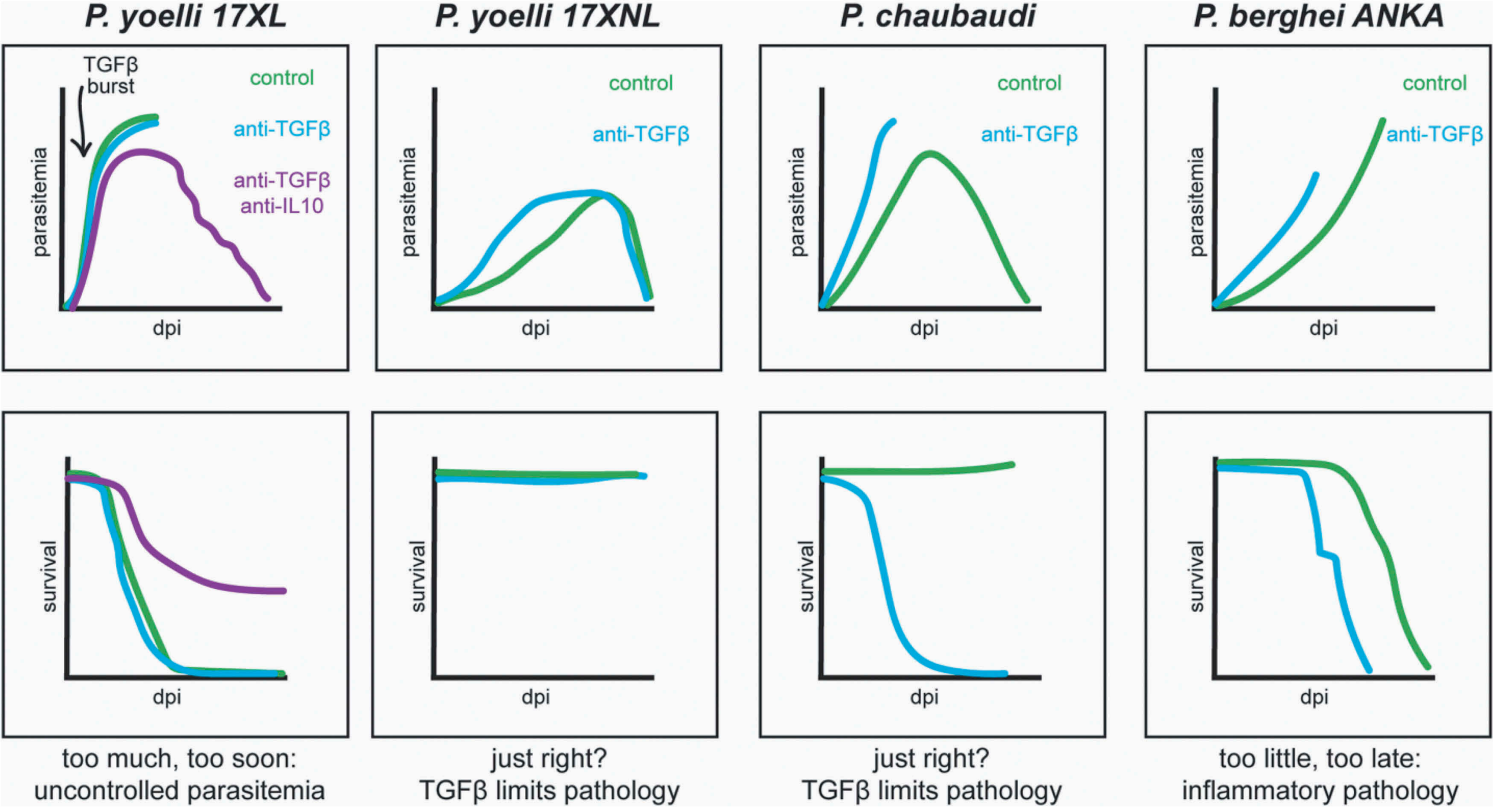
Impact of disrupting TGF-β signaling on malaria progression in murine models. dpi = days post infection. Traces compare parasitemia and survival in infected mice with TGF-β signaling disrupted by antibody neutralization (blue) or unaltered (green).

A simple interpretation of the existing data could be that TGF-β functions in a Goldilocks manner during murine malaria, in which just the right amount of TGF-β at just the right time controls immunopathology while still allowing for an effective anti-parasite inflammatory response (Figure 1). In such a Goldilocks model, infections with *P. yoelli* 17XL are lethal because the TGF-β burst observed within a day of infection is too much, too soon, and interferes with an early inflammatory response required to effectively control parasite replication [46]. When ECM is modeled with *P. berghei* ANKA, pathology and fatality occur because the TGF-β response is too little, too late, and fails to moderate the anti-parasitic inflammatory response sufficiently to limit pathology [45]. In contrast, immune responses to resolving infections with *P. chaubaudi* and *P. yoelli* 17XNL get inflammatory levels just right, with TGF-β signaling sufficiently delayed and muted to allow the anti-parasitic inflammatory response to continue at a level that controls parasitemia but does not lead to excessive pathology [17,45]. Abundant literature regarding anti–inflammatory roles for TGF-β suggests many plausible mechanisms by which TGF-β signaling could protect against pathology during severe malaria. Notably, however, the relevance of specific immunomodulatory roles for TGF-β during malaria remain untested. Establishing the mechanistic basis of TGF-β-mediated protection from malarial pathology could provide critical insights that allow for the development of therapeutic strategies that limit the pathology of severe malaria while maintaining robust parasite control.

In addition, TGF-β signaling may also contribute to the anti-parasitic response during malaria. This is suggested by multiple studies using *P. berghei* ANKA, *P. chabaudi*, and *P. yoelli* 17XNL infection models where neutralization of TGF-β hastened parasite replication kinetics (Figure 1) [45,48]. While these observations are robust, the precise mechanisms underlying enhanced parasite replication upon disruption of TGF-β signaling remain essentially unexplored. The pleiotropic nature of TGF-β signaling and complexity of the *Plasmodium* parasite lifecycle in mammalian hosts suggests a variety of modes through which TGF-β could promote parasite replication.

## Potential mechanisms of TGF-β modulation of inflammatory pathology during malaria

### Inhibiting T_H_1 and IFN-γ responses

CD4^+^ T cells are well established as both critical mediators of anti-parasitic defenses during malaria [49] and as targets for negative regulation by TGF-β [20]. CD4^+^ T_H_1 cells expand in numbers during malaria in mice and humans, and help control parasitemia during blood-stage malaria [49–51]. The protective role of CD4^+^ T cells during murine malaria is largely assumed to derive from their robust production of IFN-γ, as IFN-γ is also critical for controlling parasitemia [50,52–54]. However, CD4^+^ T_H_1 is not the only cellular subset that expresses IFN-γ during malaria. Whether the critical protective IFN-γ derives from CD4^+^ T_H_1 cells, TFH, or CD8^+^ T cells in producing protective IFN-γ during malaria remains unknown, as do the specific mechanisms by which IFN-γ contributes to parasite control [49]. Plausible mechanisms of IFN-γ-mediated protection include the promotion of macrophage activation and class switch recombination in parasite-specific B cells [49]. Despite their robust anti-parasitic effects, IFN-γ and T_H_1 T cells may also promote disease severity, as high levels of circulating IFN-γ correlate with malaria pathology in humans and mice [15,55,56]. In murine models, lack of IFN-γ protects against ECM and reduces production of pro-inflammatory cytokines [53].

TGF-β is well established as a modulator of T cell differentiation. Multiple models indicate that TGF-β inhibits differentiation of both T_H_1 and T_H_2 T cells [18]. T_H_1 expand during blood-stage malaria [57]. Accordingly, TGF-β inhibition of T_H_1 differentiation may suppress the production of T_H_1-derived IFN-γ, which could alter disease progression through the above-described effects of IFN-γ on both parasite control and malarial pathology. In contrast, T_H_2 T cells are largely absent during malaria and thus unlikely to contribute to either pathology or parasite control [49,58]. Thus, TGF-β could limit inflammatory pathology by tuning both T_H_1 differentiation and functionality. In this way, TGF-β could temper the IFN-γ response while still allowing for effective control of parasite replication during early infection.

### Promoting T-reg modulation of T effector activity

Tregs are important regulators of effector T cell activity. Tregs can be identified by their expression of the hallmark transcription factor Foxp3, and are produced both in the thymus (thymic-derived, tTregs) and periphery (peripheral, pTregs) [59,60]. TGF-β promotes development of both thymic- and peripheral-derived Tregs. Disruption of TGF-β signaling via depletion of TGF-βRI in neonatal mice does not permanently abrogate formation of tTregs, but does lead to a marked loss of Foxp3+ thymocytes in the first week of life [61]. In the case of pTregs, studies of T cells in primary cultures suggest that TGF-β promotes differentiation of CD4^+^ T cells into Foxp3-expressing Tregs [62,63], especially in the absence of strong pro-inflammatory cytokine signaling [64].

Early studies provided highly conflicting evidence as to whether Tregs promote or control parasitemia and pathology during murine malaria [65,66]. Much of this confusion likely relates to variabilities in experimental systems, particularly the infection model used and timing and method of Treg depletion. In particular, the popular early approach of using anti-CD25 treatment to deplete Tregs was plagued by retention of significant numbers of CD25^−^ Foxp3^+^ thymocytes with regulatory functionality [67]. Our group recently used the *P. yoelli* 17XNL model of infection to show that CD4^+^ Foxp3^+^ Tregs expand during a specific temporal window of blood-stage malaria. Depletion of Tregs using a Foxp3-diptheria toxin receptor (DTR) system during this temporal window lead to dramatically accelerated control of parasitemia. In contrast, earlier Treg depletion at infection onset (mimicking prior studies) resulted in mortality from this typically non-lethal infection [57]. The expansion of Tregs associated with higher parasitemia coincided with a contraction of activated CD4 T cells [57]. This suggests that the expanded Tregs enable parasite replication by interfering with the CD4 T_H_1 and TFH responses known to be critical for controlling blood stage infection. Consistent with this theory, neutralization of Tregs by Foxp3-depletion led to increased quantities of activated CD4 T cells and rapid control of parasitemia [57]. Overall, these data suggest a model where Tregs act in a discrete temporal window during blood-stage malaria, in part to limit CD4 T_H_1 responses.

If TGF-β signaling promotes Treg expansion during malaria, this could function to suppress excessive inflammation caused by unchecked CD4 T_H_1 and IFN-γ responses. At least one study has reported that TGF-β neutralization decreased the number of splenic Foxp3+ Tregs detected during *P. berghei* ANKA infection [68]. The significance of this decrease in Treg numbers remains unclear, as the functionality of these Tregs in suppressing effector T cells was not tested and the very early TGF-β neutralization scheme (prior to infection onset and continuing for the first week of infection) also substantially altered parasite replication kinetics [68]. Independent support for TGF-β promoting Treg development during malaria comes from *in vitro* studies in which co-culture of human peripheral blood mononuclear cells and RBCs infected with *P. falciparum* induced development of FoxP3-expressing Tregs, with TGF-β required for production of FoxP3^hi^ Tregs [69]. A burst of TGF-β corresponding with onset of blood-stage disease and Treg detection has also been observed in experimental human malaria infections [70]. Intriguingly, *Plasmodium* proteases released upon rupture of infected RBCs can activate TGF-β *in vitro* [71].

In total, these data could support an intriguing model where, as blood stage infection progresses, increasing quantities of parasite-derived proteases released by RBC lysis promote activation of latent TGF-β, which in turn promotes the differentiation of Tregs that act to limit inflammatory pathology. Establishing whether TGF-β is required for successful Treg expansion *in vivo* during murine malaria would greatly help to support the plausibility of this model. This model would also predict that TGF-β signaling should act in a specific window corresponding to Treg amplification to protect against pathology, a concept that is experimentally testable. Specific parasite proteases have been implicated in TGF-β activation *in vitro* [71], and could also be tested for roles in promoting the Treg expansion during murine malaria.

Notably, if TGF-β does promote Treg expansion during malaria, the impact of Treg depletion timed to correspond with contraction of CD4 T_H_1 during *P. yoelli* 17XNL infection challenges the idea that the TGF-β response is optimized to promote an optimal outcome during resolving murine malaria infections. Specifically, depletion of Tregs in this critical window dramatically improved control of parasitemia without any concomitant fatality caused by inflammatory pathology [57], suggesting that TGF-β production is not sufficiently delayed to allow for ideal control of this infection. Moreover, this study identified upregulation of the repressor of antigen-presenting cell co-stimulation CTLA-4 as a potential mediator of Treg suppression of the CD4 T cell response. Blockade of CTLA-4 not only improved control of parasitemia, but also the quality of germinal center responses and the ability to generate immunity protective against subsequent challenge with other *Plasmodial* species [57]. If CTLA-4 blockade improves these outcomes via neutralization of Treg function, this would suggest that a CD4 T_H_1 response unperturbed by TGF-β -induced Treg modulation would lead to superior functional immunity. This observation is not limited to the *P. yoelli* 17XNL model, as CTLA-4 blockade during *P. berghei* ANKA challenged also decreased mortality [57]. In combination with previous findings that TGF-β neutralization protects against mortality during *P. berghei* ANKA infection [45], this observation could be interpreted to suggest that TGF-β signaling is unlikely to promote Treg differentiation during malaria. Alternatively, TGF-β may simply exert pleiotropic effects specific to discrete temporal windows of infection, as appears to be true for Tregs.

## Potential mechanisms of TGF-β restriction of parasite growth during malaria

### Activation of naïve macrophages

Although TGF-β is most frequently discussed as anti–inflammatory cytokine, in specific circumstances TGF-β can exert pro-inflammatory effects. *In vitro* studies demonstrated that at low concentrations (0.1–10pg/mL), TGF-β functions as a chemotactic ligand for human monocytes [72]. TGF-β has also been shown to enhance transcription of the pro-inflammatory cytokines IL-1 and TNF-α in cultured human PBMCs [73]. Monocytes and their mature progeny macrophages and inflammatory dendritic cells may contribute to control of blood stage malaria by phagocytosing infected RBCs and rapidly secreting pro-inflammatory cytokines [74,75]. Macrophages also contain parasitemia by phagocytosing infected RBCs and generation of nitric oxide, and are theorized to be targets of IFN-γ activation that help explain the critical role for IFN-γ in controlling blood-stage disease [49,76,77].

Activation of innate immune cells such as monocytes and macrophages by TGF-β could explain the rapid detection of enhanced parasitemia observed upon TGF-β neutralization within 2 days of initiating blood-stage infections. Kinetics of cytokine production, including TGF-β, IL-10, IFN-γ, and TNF-α have been linked to the differential outcomes in infection with lethal and non-lethal *P. yoelli*, which starkly diverge within the first few days of infection [46]. Accordingly, it is appealing to speculate that early during murine malaria, before a robust inflammatory profile has developed, TGF-β promotes activation of anti-parasite innate effectors such as monocytes and macrophages. As parasitemia and inflammation increase, TGF-β could then transition to limiting inflammatory pathology. Whether the consequences of TGF-β neutralization differ during different phases of malaria has not been investigated, but could offer insights into the plausibility of this model.

### Promoting TFH differentiation

TFH cells provide critical help to B cells during the development of germinal center responses that are critical for resolving parasitemia during blood-stage malaria [49,78]. IL-6 promotes TFH differentiation during murine malaria [79]. Whether TGF-β also promotes TFH differentiation during murine malaria has not been tested, but TGF-β promotion of TFH differentiation has been shown in several systems including cultured human CD4^+^ T cells and in mice infected with influenza virus [80,81]. TGF-β promotion of TFH differentiation could thus help control parasitemia during the mid and late stages of primary murine malaria infections. Notably, enhanced parasitemia upon TGF-β neutralization can be observed as early as 2 days after initiation of a blood stage infection in mice [45,48], which is well before germinal centers have even formed. Thus, promotion of TFH differentiation cannot explain the very early enhancement of parasite growth upon TGF-β neutralization.

### Regulating Treg differentiation

We recently showed that in one specific temporal window, expansion in Treg numbers impaired effective control of blood-stage parasitemia [57]. However, other reports have suggested that Tregs actually promote protection against blood-stage malaria in murine models [82,83]. Moreover, in humans experimentally infected during a clinical trial, increased Treg production correlated with slower parasite growth [70]. It thus remains possible that in a different temporal window, Tregs could exert anti-parasitic effects. In this scenario, TGF-β promotion of Treg differentiation could contribute to parasite control. Alternatively, T-cell derived TGF-β has been shown to inhibit expansion of tTregs [84]. Perhaps the TGF-β activated during malaria similarly limits Treg expansion and thereby promotes better parasite control. Thus, the temporal impact of TGF-b signaling during blood-stage malaria remains unresolved.

## Perspectives and outstanding questions

Balancing anti-parasitic inflammatory responses with the need to limit severe inflammatory pathology is absolutely critical for successful resolution of malaria. The pleiotropic immunomodulatory cytokine TGF-β clearly contributes to tuning the immune response during malaria. However, the mechanisms by which TGF-β acts to prevent pathology and limit parasite replication remain a black box of unexplored possibilities.

Future studies should assess whether the function of TGF-β during malaria is temporally controlled, as has been recently been shown for immunomodulatory Tregs. Does early TGF-β signaling exert a primarily anti-parasitic inflammatory effect? Is the anti-parasitic TGF-β action mediated by previously described mechanisms of Tfh and Treg induction, or activation of naïve innate cells by TGF-β? Does later TGF-β production only limit inflammation, and if so, when does this transition occur? Would disruption of TGF-β signaling during later periods, such as Treg expansion or parasitemia resolution have similar effects as disruption during the first week of infection?

Because the effects of TGF-β signaling are highly context-dependent, further attention should be paid to the cellular sources and sensors of TGF-β during malaria. Various immune cells can produce latent TGF-β during malaria, and both host and parasite proteases may be able to activate TGF-β [71,85]. Can specific sources, sensors, or activators of TGF-β be ascribed responsibility for anti-parasitic or anti–inflammatory effects, or primary activity during specific infection stages? The notion that *Plasmodium* parasite-derived proteases may directly contribute to the activation of latent TGF-β is intriguing. Does parasite-mediated TGF-β activation occur *in vivo*, and what is its functional significance?

In many systems, TGF-β is a critical promoter of Treg differentiation. Co-culture systems suggest TGF-β-dependent parasite-induced Treg differentiation is at least possible *in vitro*. However, the role of Tregs in malaria is only partially understood. The best evidence so far suggests that Tregs suppress optimal anti-parasite immunity. This contrasts with the primarily anti–inflammatory effects of TGF-β during murine malaria. What are the critical signals that trigger *in vivo* expansion of Tregs during malaria, and is TGF-β among them?

Filling in the black box of TGF-β activity during malaria will help to define the parameters by which anti-malarial immunity can effectively constrain parasite replication while minimizing inflammatory pathology.
